# Recurrent aseptic meningitis in association with Kikuchi-Fujimoto disease: case report and literature review

**DOI:** 10.1186/1471-2377-12-112

**Published:** 2012-09-29

**Authors:** Tomoko Komagamine, Takahide Nagashima, Masaru Kojima, Norito Kokubun, Toshiki Nakamura, Kenich Hashimoto, Kazuhito Kimoto, Koichi Hirata

**Affiliations:** 1 Department of Neurology, Dokkyo Medical University, 880 Kitakobayashi, Mibu, Shimotsuga, Tochigi, 321-0293, Japan; 2 Department of Anatomic and Diagnostic Pathology, Dokkyo Medical University, Tochigi, Japan; 3 Department of Neurology, Amakusa Rehabilitation Hospital, Saitama, Japan; 4 Department of Neurology, Keiju General Hospital, Ishikawa, Japan

**Keywords:** Recurrent aseptic meningitis, Kikuchi-Fujimoto disease, Histiocytic necrotising lymphadenitis, SLE

## Abstract

**Background:**

Kikuchi Fujimoto disease (KFD), or histiocytic necrotising lymphadenitis, is a benign and self-limiting condition characterised by primarily affecting the cervical lymph nodes. Recurrent aseptic meningitis in association with KFD is extremely rare and remains a diagnostic challenge.

**Case presentation:**

We report a 28-year-old man who presented 7 episodes of aseptic meningitis associated with KFD over the course of 7 years. Histopathological findings of enlarged lymph nodes led to the diagnosis of KFD. The patient’s headache and lymphadenopathy spontaneously resolved without any sequelae.

**Conclusions:**

A diagnosis of KFD should be considered when enlarged cervical lymph nodes are observed in patients with recurrent aseptic meningitis. A long-term prognosis remains uncertain, and careful follow-up is preferred.

## Background

Kikuchi-Fujimoto disease (KFD), or histiocytic necrotising lymphadenitis, is recognised as a benign lymphadenopathy that has acute or sub-acute onset and is primarily localised within the cervical lymph nodes. KFD has various extranodal manifestations, including skin lesions, gastrointestinal symptoms or splenomegaly [1]. Neurological complications, including aseptic meningitis, mononeuritis multiplex or acute cerebellar ataxia, are not common [2], and a meta-analysis of 244 KFD cases in 181 published case reports demonstrated 11% of incidence of neurological involvements [3]. The most common neurological complication is aseptic meningitis, which is observed in 2.8-9.8% of KFD cases [4,5]. KFD usually resolves spontaneously within a few months, and the recurrence rate is 3-4% [6]. Recurrent aseptic meningitis associated with KFD is an extremely rare condition, and only 4 sporadic cases have been reported [7-10]. In this study, we describe a KFD patient who presented with 7 episodes of recurrent meningitis. We also investigate the clinical and laboratory features of 4 patients previously reported in the literature.

## Case presentation

**Figure 1:**
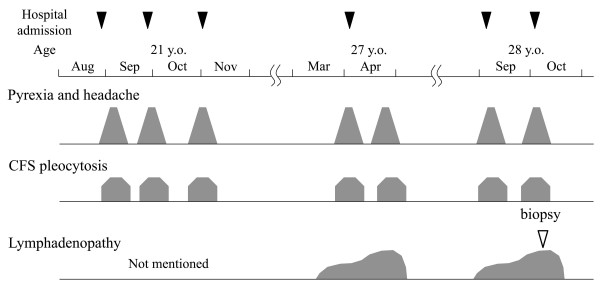
**Clinical course of the patient.** The patient had his first meningitis episode at 21 years of age. Two resolutions and exacerbations occurred within the following 3 months. He had the fourth to seventh episodes of meningitis between 27 and 28 years of age. In each episode the patient’s symptoms and abnormal cerebrospinal fluids were spontaneously resolved. Lymphadenopathy was evident both in the attacks at age 27 and age 28.

A 28-year-old Japanese man was admitted to our hospital because of headache and remittent fever that had lasted for 12 days after a solar exposure. The patient had a history of atopic dermatitis from age 2 and had 5 previous episodes of aseptic meningitis with an undetermined aetiology between the ages of 21 and 27 years-old (Figure 1).

Upon admission, day 12, the patient was alert and had pyrexia of 39°C and severe headache with positive Kernig's Sign. The palpable tender lymph nodes with 10 mm in size were present on the right posterior neck, similar to a recent meningitis episode. No other neurological deficits were noted. Laboratory analysis revealed no abnormalities in the patient’s complete blood cell count or liver and thyroid function tests. His serum CRP and IgE levels were elevated, measuring 2.5 mg/dL (normal, <0.3) and 6950 mg/dL (normal, < 295), respectively. The cerebrospinal fluid (CSF) examination showed a crystal clear appearance and pleocytosis of 27 cells/mm^3^ (97% mononuclear cells) with a protein concentration of 31 mg/dL (normal, < 45). The CSF glucose/glycaemia ratio was 0.86 with sterile bacterial, tuberculosis and fungal cultures. A CSF polymerase chain reaction (PCR) assay for the herpes simplex virus (HSV) was negative. The patient recovered after the administration of non-steroidal anti-inflammatory drugs as a symptomatic treatment and was discharged in remission on day 26.

Subsequently, the patient was re-admitted on day 37 with headache and remittent fever. He was febrile with re-appearance of Kernig's Sign. The posterior cervical lymph nodes were enlarged and tender bilaterally. Additionally, the patient also had oral aphthae and skin rashes on his trunk along with atopic dermatitis. He was alert, and no focal neurological deficit was noted. A complete blood count revealed leukocytopaenia (2.7 x 10^9^/L) with 1% of atypical lymphocytes. A biochemical examination showed abnormal levels with LDH 729 U/L (normal, <220), ferritin 2660 ng/mL (normal, 25–280) and an erythrocyte sedimentation rate of 59 mm/h (normal, <10). IgE and CRP assays exhibited higher levels than those of previous tests, with values of 10400 mg/dL and CRP 6.5 mg/dL being observed, respectively. Serum anti-nuclear and anti-neutrophil cytoplasmic antibodies and rheumatoid factor were within normal range. The complement 3 level was slightly elevated with 194 mg/dL (normal, 65–135). Human leukocyte antigen B51 was negative. The evidence of other infectious agents, such as human hepatitis viruses B and C, human T-cell lymphoma virus-1, syphilis, HSV-1 and 2, varicella-zoster virus (VZV), cytomegalovirus, Epstein-Barr virus (EBV), human herpes virus type 6 (HHV-6) and toxoplasmosis (*Toxoplasma gondii)* were not detected. A CSF examination revealed a pleocytosis of 16 cells/mm^3^ (97% mononuclear cells) and a protein concentration of 28 mg/dL, as well as a CSF glucose/glycaemia ratio of 0.75 with sterile bacterial, tuberculosis and fungal cultures. CSF PCRs for HSV, HHV-6, VZV and tuberculosis were negative. The patient’s CSF IgE level was not elevated (19 IU/ml, IgE index 0.03). Splenomegaly was present upon abdominal echograph. No enlargement of deep lymph nodes was detected using computed tomography. Moreover, brain magnetic resonance imaging showed no structural abnormalities.

**Figure 2:**
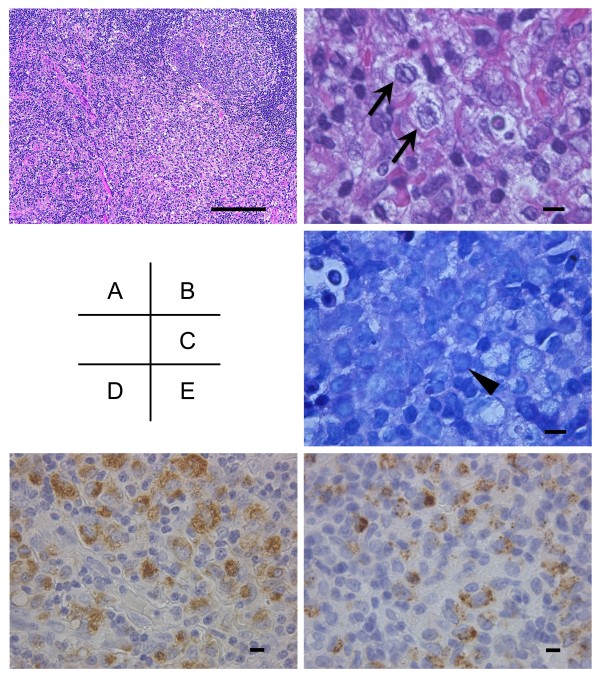
**Histopathological findings of affected lymph node. A**: In a low-power field, the affected lymph node showed discreet areas of necrosis paracortex (Hematoxylin-eosin stain, 10X). **B**: In a high-power field, the lesion demonstrated abundant karyorrhectic debris with apoptosis bodies and numerous histiocytes and large lymphoid cells (arrows). There were no neutrophils in the lesion (Hematoxylin-eosin stain, 100X). **C**: Giemsa stained section highlighted the plasmacytoid dendritic cells cluster (arrowheads) at the margins of the necrotic foci (may-Giemsa stain, 100X). **D**: Immunostain demonstrated that many histiocytes in the necrotic area were positive for myeloperoxidase (60X). **E**: Portions of the hystiocytes were stained with CD68 (60X). The scale bars in each panel indicate 10 μm, except for panel A (50 μm).

An excisional biopsy of the involved posterior cervical lymph nodes was performed. The affected lymph nodes showed focal paracortical necrotic lesions (Figure 2A). Under a high power field, the lesion had abundant karyorrhectic debris with apoptotic bodies, numerous histiocytes and large lymphoid cells and scattered fibrin (Figure 2B). However, there were no neutrophils in the lesion. A portion of the phagocytic macrophage had crescent nuclei. Moreover, Giemsa stained sections highlighted the plasmacytoid dendritic cells clusters at the margins of the necrotic foci (Figure 2C). Immunohistochemical study demonstrated that the histiocytes expressed CD68 and myeloperoxidase (Figure 2D, E). Based on these pathological findings, the diagnosis of KFD was made.

The patient did not receive medication during his second admission. Also, his symptoms and CSF parameters spontaneously resolved within two weeks. His enlarged lymph nodes gradually decreased in size. The patient was discharged in remission on day 49, and he remains headache-free after 28 months of follow-up.

## Discussion

Since KFD was first described by both a pathologist and physician independently in 1972 [11,12], the aetiology of KFD remains largely unknown. The histopathological features of affected lymph nodes in KFD are, on occasion, notably similar to those of SLE [13]. Therefore, pathogenic linkage between the two disorders has been proposed [1,13]. Infectious agents, including toxoplasmosis, EBV, and HHV-6, have also been considered as possible causal agents, but several studies have failed to confirm their association [13,14]. There is no specific treatment for KFD because of its unknown aetiology. In general, the patients are treated symptomatically; for example, relief of local and systemic complaints with the use of analgesics, antipyretics and rest [13]. Furthermore, corticosteroids may be effective in severe cases or for a relapsing condition [1].

The aseptic meningitis associated with KFD was first reported in 1979 [4]. Today, 18 sporadic case reports have been documented in MEDLINE and Japan Medical Abstracts Society-website, with 4 of them reporting recurrence of meningitis [7-10]. The clinical profiles of our patient and the 4 patients reviewed in the literature are shown in Table 1. In all 5 cases the symptoms resolved within several months. Corticosteroids were administered in 3 out of the 5 patients. All of the 3 did not have early post-treatment relapse after receiving steroids. Steroid treatment may be beneficial for recurrent KFD with aseptic meningitis, although recommendation of steroid administration requires further investigation. 

**Table 1:** **The clinical features of recurrent aseptic meningitis cases with Kikuchi-Fujimoto disease**

**Case and reference number**	**1**[7]	**2**[8]	**3**[9]	**4**[10]	**5 (present case)**
Age/gender	21/M	12/F	29/M	35/F	28/M
Meningeal sign	-	+	-	+	+
Maximum Cerebrospinal fluid cell count/mm^3^	178	100	63	59	137
Duration of the meningitis episode	10 days	10 days	10-30 days	10 days	10 – 20 days
Interval between each episode	1 month	1 week	11 years	5 months, 11 months	1 week to 6 years
Coexisting conditions	High titre of anti-Toxoplasma antibody	-	Elevation of serum IgE	High titre of antinuclear antibody	Elevation of serum IgE
Steroid Use	-	Oral prednisolone,	Intravenous methyl prednisolone,	Oral prednisolone,	-
		1 mg/kg		40 mg/day	
			1000 mg/day		

Our patient had concurrent atopic dermatitis, and his serum IgE levels were elevated along with exacerbation from meningitis and lymphadenitis. Because IgE was not elevated in the CSF, his high serum IgE titre did not appear to play a pathogenic role in aseptic meningitis. A prior case with an elevation of serum IgE in recurrent aseptic meningitis with KFD has been reported [9]. In this study, we speculate that IgE elevation may be reflected the immunostimulatory condition that was activated upon KFD in a patient with atopic dermatitis. A striking histopathological feature of KFD is the clustering of the plasmacytoid dendritic cells at the margins of the necrotic foci of affected lymph node [1]. Plasmacytoid dendritic cells are known to produce type I interferon in response to viral infection and to induce human memory B cells to differentiate into plasma cells and produce immunoglobulin [15]. Type I interferon is known as a potential pathogenic agent in the SLE-related neurological involvement [16]. Moreover, the high titre of antinuclear antibodies had also been observed in another case [10]. The progression of recurrent aseptic meningitis with KFD may stem from pathogenic association with SLE or other autoimmune disorders. Further studies are necessary to clarify this hypothesis.

## Conclusions

Recurrent aseptic meningitis with KFD is extremely rare condition. However, awareness of KFD as the differential diagnosis for meningitis might assist with diagnosis of patients presenting with lymphadenopathy. Early excisional lymph node biopsy should be considered to avoid unnecessary treatments. Temporary corticosteroid treatment may be beneficial to patients that present with recurrent meningitis with KFD, although this treatment’s long-term efficacy remains uncertain. Because of the association with SLE, patient follow-up visit is necessary upon subsequent development of symptoms.

### Consent

Written informed consent was obtained from the patient for the publication of this case report.

### Ethics approval

The study was approved by the Human Ethics Review Committee of Dokkyo Medical University.

## Abbreviations

CSF: Cerebrospinal fluid; EBV: Epstein-Barr virus; HHV-6: Human herpes virus type 6; HSV: Herpes simplex virus; KFD: Kikuchi Fujimoto disease; PCR: Polymerase chain reaction; SLE: Systemic lupus erythematosus; VZV: Varicella-zoster virus.

## Competing interest

The authors declare that they have no competing of interests.

## Authors’ contributions

KT and NT had full access to all of the data in the study and take responsibility for the integrity of the data and the accuracy of the data analysis. Study concept, design, and interpretation: KT and NT. Collection and analysis of data: KT, NT, KK and HK. Critical revision of the manuscript: NT and KN. Pathological interpretation: KM. Figure arrangement: KN and KT. All authors read and approved the final manuscript.

## Pre-publication history

The pre-publication history for this paper can be accessed here:

http://www.biomedcentral.com/1471-2377/12/112/prepub
